# Autochthonous Wheat Grown in Organic and Conventional Systems: Nutritional Quality of Flour and Bread

**DOI:** 10.3390/foods13071120

**Published:** 2024-04-07

**Authors:** Nerea Fernández-Canto, María Belén García-Gómez, María Lourdes Vázquez-Odériz, Matilde Lombardero-Fernández, Santiago Pereira-Lorenzo, Ángel Cobos, Olga Díaz, María Ángeles Romero-Rodríguez

**Affiliations:** 1 Areas of Nutrition and Food Science and Food Technology, Department of Analytical Chemistry, Nutrition and Food Science, Faculty of Sciences, Campus Terra, Universidade de Santiago de Compostela, 27002 Lugo, Spain; marianerea.fernandez.canto@usc.es (N.F.-C.); mariabelen.garcia@usc.es (M.B.G.-G.); lourdes.vazquez@usc.es (M.L.V.-O.); angel.cobos@usc.es (Á.C.); angeles.romero@usc.es (M.Á.R.-R.); 2 Agronomy and Animal Science Group, Department of Anatomy, Animal Production and Veterinary Clinical Sciences, Campus Terra, Universidade de Santiago de Compostela, 27002 Lugo, Spain; matilde.lombardero@usc.es; 3 Instituto de Biodiversidade Agraria e Desenvolvemento Rural (IBADER), Campus Terra, Universidade de Santiago de Compostela, 27002 Lugo, Spain; santiago.pereira.lorenzo@usc.es; 4 Department of Plant Production and Engineering Projects, Escuela Politécnica Superior, Campus Terra, Universidade de Santiago de Compostela, 27002 Lugo, Spain

**Keywords:** mineral, PGI Pan Gallego, landrace, cereal, protein, starch

## Abstract

A growing interest in the recovery and enhancement of crops, particularly local varieties such as ‘Caaveiro’ wheat, has been observed. This study aims to investigate the impact of cultivation systems (organic versus conventional) on the nutritional quality of ‘Caaveiro’ flour and breads protected by the PGI “Pan Galego,” employing two fermentation methods (sourdough versus sourdough and biological yeast). Organic flour exhibited significantly higher levels of moisture, fat, sucrose, phosphorus (P), sodium (Na), and copper (Cu) while also exhibiting a lower total starch and zinc (Zn) content. Organic bread, produced using both fermentation methods, demonstrated significantly higher protein, carbohydrate, total, resistant, and rapidly digestible starch, ash, Na, P, iron (Fe), and Cu content. Additionally, they contained less moisture compared to conventional bread. Despite variations in nutritional characteristics based on the cultivation system, the organic approach proved effective at producing high-quality products with a positive environmental impact, which is highly appreciated by consumers.

## 1. Introduction

Wheat is one of the most widely used cereals in breadmaking because it has better breadmaking characteristics compared to other cereals [1]. In addition, nearly 80% of the daily diet in many developing countries consists of wheat bakery products, making bread one of the most consumed products in the world [2]. 

In Galicia (NW Spain), the Protected Geographical Indication (PGI) ‘Pan Galego’ was recently created [3]. Galician bread is a traditional oven-baked product characterized by its artisanal method of production using soft wheat flour (*Triticum aestivum*, L.). According to the specifications of the PGI, 25% of this flour must come from local Galician varieties of wheat (‘Caaveiro’ and/or ‘Callobre’), while the remaining 75% may be sourced from other foreign varieties of wheat flour. In addition to flour, the other ingredients used should include water (at least 75 L per 100 kg of flour), sourdough (at least 150 g/kg of flour weight), and salt. Optionally, biological yeast can be added (maximum 15 g/kg of flour). The elaboration process requires a minimum of a three-hour fermentation time and for the loaves to be baked in stone ovens.

It Is precisely the steps involved in this breadmaking process, including the high degree of hydration, the incorporation of autochthonous wheat varieties, the use of sourdough as a leavening agent, the long fermentation times, and the use of stone ovens for baking that provides ‘Pan Galego’ (Galician bread) with its own sensory characteristics which, in turn, means that this product is highly appreciated at a national level [4]. 

In Galicia, there are initiatives to recover local wheat varieties, as was recently demonstrated with the ‘Caaveiro’ and ‘Callobre’ varieties. Promoting the recovery of local varieties is an important endeavor since they are more resistant to the propagation of pathogens, are better adapted to the environment [5], and because they require fewer inputs [6]. In addition, when commercial varieties were compared to varieties of autochthonous wheat, Arzani & Ashraf [1] indicated that the commercial varieties studied showed lower nutritional quality, as seen in the lower content of minerals and other micronutrients. With the recovery of local varieties, the loss of genetic diversity is avoided, and the recovery of traditional agricultural practices is promoted. These initiatives are frequently oriented towards organic production or the elaboration of artisanal products of marked quality [5].

Products derived from wheat, including bread, are an important source of energy, which mainly comes from complex carbohydrates such as starch. In addition, these products provide protein, fiber, and micronutrients, such as minerals, B vitamins, antioxidants, and phytochemicals [7]. From a nutritional point of view, the digestibility of starch should be taken into account since it is a carbohydrate with a high glycemic index that is related to obesity and other metabolic diseases [8]. 

Starch is classified according to its digestibility in the upper digestive tract and the rate and degree of digestive enzyme hydrolysis. Starch digestibility classifications are divided into fractions. Fraction (a) is rapidly digestible starch (RDS) and one that is converted to constituent glucose molecules within 20 min of enzyme digestion. This starch may promote a metabolic syndrome that can result in insulin resistance, obesity, and diabetes. Fraction (b) is slowly digestible starch (SDS), which is completely digested in the small intestine from 20 to 120 min after ingestion. The benefit of this starch is its moderate impact on the glycemic index. Fraction (c) is resistant starch (RS) that is not digested by human enzymes in the small intestine and may be fermented by colonic microflora in the large intestine [8,9]. RS is the portion of starch that is not absorbed in the small intestine of healthy humans [10] and passes to the large intestine to perform physiological functions like those of dietary fibers. By contrast, a high RDS value for starch indicates that it is rapidly hydrolyzed in the small intestine and is associated with high and unstable blood glucose levels. Therefore, prolonged consumption of foods with high RDS can result in type 2 diabetes, obesity, and coronary heart disease [11]. Consequently, SDS and RS are the most nutritionally interesting fractions.

Recently, a study in which ancient wheat varieties were shown to be connected to better nutritional parameters in terms of metabolic diseases, such as the glycemic index, which may be related to starch fractions, was published [1]. Although another recent investigation found no differences between ancient and modern wheat varieties, their digestibility might be more related to the breadmaking process [8].

In recent years, there has been an increased demand for higher-quality products obtained in a sustainable way. Thus, in Europe, the market for organic products has begun to be more prevalent; indeed, it increased by 8.28% from 2019 to 2020 [12,13], and in Spain increased a bit lower, by 7% [14]. 

The consumer perceives organic products as healthier, safer, and more environmentally friendly. The preference of consumers for organic foods is based on the association of these products with the concept of health because the consumer believes that these products have higher nutritional value and are free from agrochemical residues [15]. In addition, consumers who choose this type of product are more aware of environmental sustainability and protection, and they consider that the production of ecological products favors the development of rural areas [16]. 

In a review of 343 articles, Barański et al. [17] affirmed that plant foods from organic farming have higher concentrations of carbohydrates but lower concentrations of protein and fiber. Brantsæter et al. [18] obtained similar results and, in addition, sustained that organic plant products contain higher concentrations of dry matter and certain minerals (Ca, Mg, P, and K). Hurtado-Barroso et al. [19] affirmed that the organic cultivation of vegetables and fruits can be positively related to human health since products from organic cultivation have greater amounts of phenols, carotenoids, and vitamin C. These authors associate organic management with avoiding the use of pesticides, which, in turn, favors the production of natural defense substances such as polyphenols in response to environmental stress. 

In their literature review of different plant foods grown organically and conventionally, Bernacchia et al. [20] concluded that the results are highly variable and that the differences are insignificant; therefore, there is no clear evidence that the production system affects the nutritional quality of the products.

Nevertheless, different authors affirm that most comparative studies do not take into account various factors that influence the nutrient content of foods, such as variety, climatic conditions, or the type of practices used in the cultivation system [19,20]. Therefore, it is necessary to consider these factors when comparing organic versus conventional products.

Although there is scientific evidence of the environmental benefits of organic wheat cultivation [6], studies that evaluate the nutritional quality of organic vs. conventional wheat are scarce and inconclusive. Most of these studies focus on the analysis of some nutritional parameters such as protein, mineral, or moisture content [21,22,23]. In addition, there are no comparative studies between organic vs. conventional bread at a nutritional level since they compare the flour and not the final product without taking into account the effect of processing on nutritional quality [20]. Therefore, the objective of this study is to compare, at a nutritional level, local Galician ‘Caaveiro’ wheat flours obtained through organic and conventional farming practices and the bread obtained with these flours using sourdough or a mixed method (sourdough and biological yeast) as the leavening agent, which are both legally allowed in the production of the PGI ‘Pan Galego’. 

## 2. Materials and Methods

### 2.1. Samples

The ‘Caaveiro’ local wheat flour used in this study was supplied by the Da Cunha company and came from two production systems: organic and conventional. Elemental subplots of 10 × 6 m (60 m^2^) were designed. These plots were in northwestern Spain (43°15′20″ N, 8°21′18″ W). A completely randomized block design was conducted. The type of crop (organic vs. conventional) was evaluated with 4 repetitions per crop in each type of crop. In total, 8 subplots were cultivated (2 types of cultivation × 4 repetitions). 

The differences in organic and conventional wheat cultivation are related to the amount and type of herbicide. In the conventional subplots, a pre-emergence herbicide was applied (pendimethalin 40% + gliphosate 36%). The post-emergence phytosanitary treatment was carried out with prosulfocarb 80% + diflufenican 50% + clorsulfuron 75%. In the organic plots, no herbicide treatment was applied. To evaluate the nutritional characteristics of the wheat grain, the samples were collected and ground into flour (average particle size of 220 μm) in a stone mill (mod. MGH, Técnica de Mantenimiento, Lugo, Spain). 

Four types of bread were prepared in accordance with the requirements of the Protected Geographical Indication ‘Pan Galego’ [3] (organic vs. conventional). Using both conventional and organic flours, two types of bread were made, one with sourdough and the other with a mixed method (sourdough and biological yeast, that is fresh commercial yeasts of the species *Saccharomyces cerevisiae*, commonly used as a leavening agent for making bread and bakery products). Thus, it is possible to compare the cultivation method and the two fermentation methods allowed by the PGI ‘Pan Galego’. Breads were prepared in a traditional bakery, under our supervision, using 100% ‘Caaveiro’ flour per 100 kg of flour/80 L of water, 30 kg of sourdough or 30 kg of sourdough and 0.5 kg of biological yeast (mixed method) and salt. It was kneaded for 1.5 h and left to ferment for 3 h (1.5 h in hopper fermentation and 1.5 h in ball-shaped pieces). Baking was carried out in a stone oven for 1 h at 220° ± 5 °C. The bread was shaped like a bun and weighed 1.2 kg. The process was performed under the same conditions for flours from both cultivation systems (organic and conventional) and both fermentation systems (sourdough and mixed method). The processing conditions were in accordance with the specifications of the PGI Galician bread [3]. 

### 2.2. Nutritional Analysis

The samples of flour and bread were analyzed for moisture, protein, ash, fat, fiber, starch, carbohydrates, sugars, and mineral contents. To determine moisture, the samples were dried according to the official AOAC method 925.10 [24]. The crude protein was evaluated by the Kjeldahl method (N × 5.7) using an automatic distillation (based on AOAC 920.87) [24], ash content was determined by incineration at 550 ± 15 °C (method 923.03) [24], and the crude fat was determined using a Soxhlet apparatus by extracting a known weight of the powdered sample with petroleum ether and diethyl ether. The total carbohydrate content was obtained by difference. The analyses were performed in triplicate. 

The content of total fiber in bread was measured using the Megazyme total dietary fiber analysis kit K-TDFR-100A (Megazyme International Ireland Ltd., Wicklow, Ireland) based on AOAC 985.29 [24]. Additional determinations of glucose, fructose, and sucrose were performed using the Boehringer–Mannheim enzymatic kit reference number 10716260035. The content of total starch (TS) was determined in flour samples using a total starch assay kit K-TSTA-100A. The contents of TS (total starch), RDS (rapidly digestible starch), SDS (slowly digestible starch), and RS (resistant starch) were determined in bread samples with the digestible and resistant starch assay kit (K-DSTRS) (Megazyme International Ireland Ltd., Wicklow, Ireland). The analyses were performed in duplicate. 

The mineral content was determined by an ICP-MS (Agilent 7700x, Madrid, Spain) with a sample introduction system consisting of a Micromist glass low-flow nebulizer, a double-pass glass spray chamber with a Peltier system (2 °C) and a quartz torch. Measurements were made in quintuple. 

The calibration standards were prepared in NO_3_H-H_2_O, in the same proportion as in the samples. As calibration standards, the multi-elemental standard Multi IV (Merck^®^, Madrid, Spain) in concentrations between 0.2 and 10,000 µg·L^−1^, the P and Ca standard (Panreac^®^, Barcelona, Spain) in concentrations between 0.1 and 10 mg·L^−1^ and Selenium (Merck^®^) in concentrations between 0.2 and 100 µg·L^−1^ were used. The correlation coefficients of the calibration lines for each element were equal to or greater than 0.999. All results were reported on a wet-matter basis.

### 2.3. Statistical Analysis 

Mean and standard deviations were calculated. The t-Student’s test was performed to establish whether there were statistically significant differences between the sample means. The statistical significance level was set at *p* < 0.05.

All calculations were performed with IBM SPSS Statistics for Windows version 25 (IBM Corp., Armonk, NY, USA). The PCA and correlation graphs between the mineral content were performed with the R packages FactoMineR and ggplot2, respectively.

## 3. Results and Discussion

### 3.1. Flour Results 

**Figure 1:**
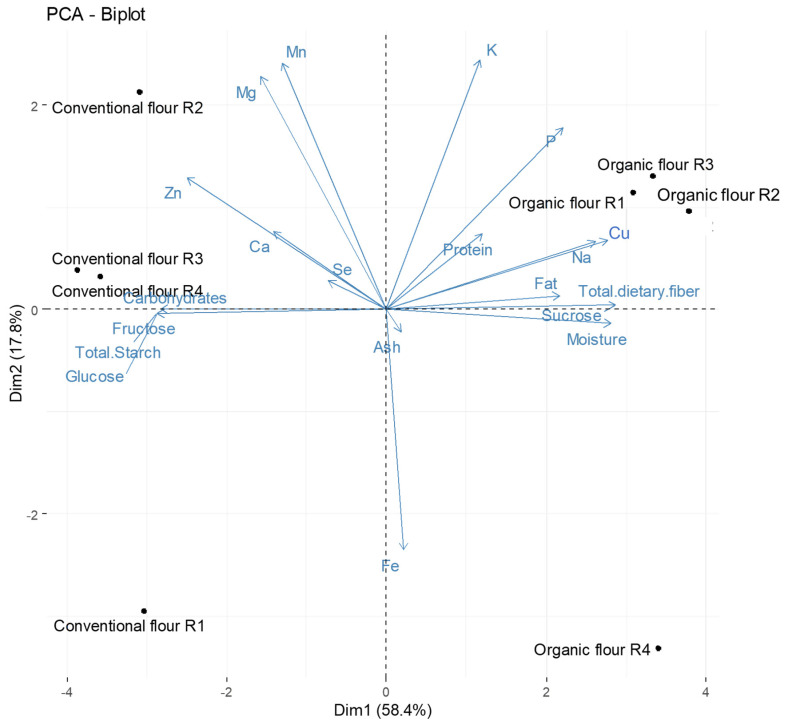
Principal component analysis (PC1 vs. PC2) describing the nutritional variation among the flours.

With the data obtained after the analysis of the flour samples, the principal components analysis was carried out (Figure 1). In total, 58.4% of the total variance was represented in the first principal component (PC1), while principal component two (PC2) explained up to 17.8% of the total variance.

In the two-dimensional space, it was possible to observe the clearly differentiated behavior between the organic and conventional flours. Considering the positions of the samples in the two components, differentiated behaviors were observed between the flour samples depending on their type of cultivation. According to PCA (Figure 1), the organic flour samples had higher moisture, protein, fat, sucrose, fiber, P, Na, Cu, and lower total carbohydrate, glucose, fructose, TS, Ca, and Zn than conventional flours. Due to the fact that the flours from the different organic plots and the conventional plots presented the same behavior, the means of the flour results are shown in Table 1.

Table 1 summarizes the results obtained in the flour. The cultivation system significantly affected the moisture, fat, total starch, and sucrose. No significant differences were found for the rest of the parameters (ash, protein, carbohydrates, glucose, fructose, and fiber).

The flour from grains that were grown under conventional conditions had a lower average moisture content (12.70 ± 0.14 g/100 g) than that produced organically (13.26 ± 0.24 g/100 g). Nitika et al. [25] also reported higher moisture values in organic cultivation (12.9 g/100 g) than in conventional conditions (10.9 g/100 g) when they studied five varieties of wheat grown in India. However, other authors indicated that there were no differences between organic and conventional flours in this parameter [26]. 

In the present study, it was observed that organic flour had a higher fat content (1.65 ± 0.25 g/100 g) than conventional flour (1.31 ± 0.23 g/100 g). A similar behavior was observed by Nitika et al. [25], where the wheat grown under organic conditions had a significantly higher fat content than the wheat grown under conventional conditions. These values were similar to those of the present study and also to other traditional varieties, such as Macedonian landraces of bread wheat [7].

Regarding simple sugars, only sucrose was significantly affected by the cultivation system, with organic flours presenting higher values than conventional ones. Zörb et al. [27] analyzed the sugar in the grains and ears of wheat obtained in Switzerland using two conventional systems (one used a mineral fertilizer and another employed mineral fertilizer plus farmyard manure) and two organic systems (biodynamic and bioorganic); they found no differences as per the system of cultivation. 

Organic flours contained a lower amount of total starch (66.50 ± 0.16 g/100 g) than conventional flours (67.40 ± 0.20 g/100 g). These results differed from those reported by Ceseviciene et al. [28] when they analyzed the grains of four Lithuanian winter wheat varieties since the organic varieties in that study showed a slightly higher content.

No significant differences were found in the fiber content in relation to the cultivation system. The fiber concentration of the flour varies depending on the degree of extraction and the grinding process. 

The ash content was similar in the flour produced with the two farming methods. These results agreed with those previously reported by Nitika et al. [25], where there were no differences depending on the system. Park et al. [22] reported significant differences in ash content depending on the cultivation system, although without observing a clear trend. This study concluded that the genotype of the varieties had more influence than the production system. The ash content found in the present article was four times lower than that reported by Nitika et al. [25]. This difference was most likely due to the fact that these authors analyzed whole meal flours, which presented higher values of mineral concentration [29].

Protein influences the quality of wheat and is a good indicator of baking [28]. However, it is not only the amount of protein but also the quality, especially that of gliadin and glutenin, which form the gluten network [26]. No significant differences were found in the protein content of the flours depending on the cultivation system, which indicates that it is possible to obtain the same amount of protein in the flours using organic practices. These results agreed with those obtained in a previous study [22], although there were other studies that reported significant differences in the protein content depending on the cultivation system. Some authors indicated that the flours that come from conventional cultivation have higher protein contents [25,26,27,28], and other authors indicate the opposite [30]. 

**Figure 2:**
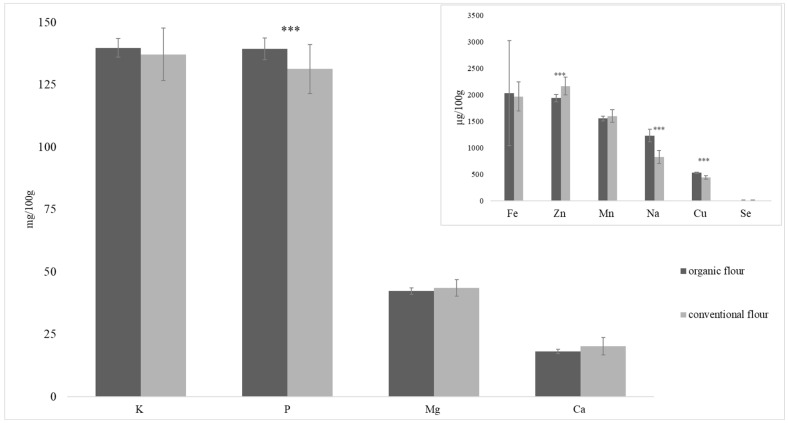
Mineral content of flour from organically and conventionally grown wheat (* *p*-value < 0.05, ** *p*-value < 0.01 and *** *p*-value < 0.001).

The mineral content of flour has been analyzed. The results obtained for flour are shown in Figure 2. The trend of the average elemental concentration in flours is as follows: K > P > Mg > Ca > Fe ≃ Zn > Mn > Na > Cu > Se. These organic flours have a higher P, Na, and Cu content and lower Zn content than conventional flours.

Vrček et al. [23] also reported lower Zn levels in wheat cultivated using organic fertilizers compared to conventional systems. They found differences during the two years of the study in K, Zn, Mo, Ca, Mn, and Fe.

Park et al. [22] studied the mineral content of flour from wheat grown using organic and conventional systems. There were differences in certain mineral elements, although there was no trend associated with the cultivation system; instead, it depended on other factors such as the variety studied, the year of cultivation, and the level of fertilization.

Ryan et al. [21] also obtained higher Cu and P values in organic than in conventional wheat in a study carried out in Australia, although the Zn content was higher with organic farming methods than with conventional ones. The trend for P content is that it is higher in organic crops due to the use of fertilizers containing soluble P [21]. 

In the data presented by Nitika et al. [25], there were significantly higher values for Cu, P, Mg, and Mn in five wheat varieties grown under no organic conditions. Nelson et al. [30] studied five western Canadian spring wheat cultivars in two farming systems. They reported higher Zn, Mg, K, and Fe values for organic farming systems but lower Se and Cu. 

Different studies reported values of P, Mg, K, Ca, Fe, and Mn that were two to three times higher than the values obtained in the present study [21,22,23,25,30]. This may be due to the fact that these authors analyzed wholemeal flour and the flour analyzed in the present study presented s more refined flour. The degree of extraction of flour had a major impact on the resulting mineral content. The mineral nutrients could be 2–5 times higher in wholegrain than white flour [31]. 

In a recent study, Wang et al. [31] compared the effect of different wheat species (*Triticum aestivum* vs. *T. spelta*), farming systems (organic vs. conventional), and flour types (wholegrain vs. white) and found that the content of P, K, Mg, Mn, Zn, Cu, and Fe was higher in organic flours than in conventional ones. No study has been found that compares the mineral content under the same conditions (variety, year, soil characteristics, etc.) and for different farming methods (organic vs. conventional).

**Figure 3:**
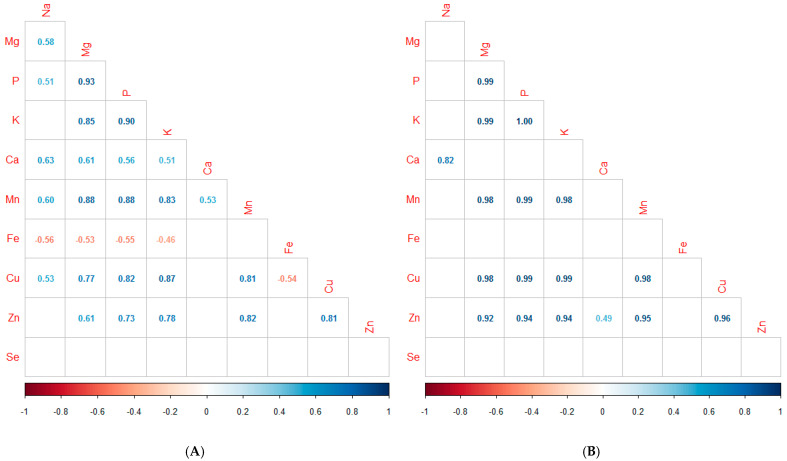
Pearson correlation coefficients among mineral concentrations of organic flour (**A**) and conventional flour (**B**). The red and blue colors, respectively, exhibit positive and negative correlations between different indexes. Non-significant correlations were omitted.

The correlation coefficients among the 10 minerals analyzed in the flours are provided in Figure 3. In general, both flours follow the same trend regarding the correlation of minerals, although the correlation coefficients are higher in conventional flours. With the exception of Se, all the minerals of the organic flours presented significant correlations (Figure 3A). The highest correlations in these flours were found between P and Mg (r = 0.93), K and P (r = 0.90), Mn and Mg (r = 0.88), Mn and P (r = 0.88) and K and Mg (r = 0.85). 

In conventional flours (Figure 3B), there were also significant positive correlations between the content of Mg, P, and K with Mn, Cu, and Zn, between the contents of K and P or Mg, and between Mn and Cu or Zn. However, no correlations were found between Zn with Na or Ca.

Gomez-Becerra et al. [32] also found positive correlations between P and Mg or Zn. These strong associations between P and Zn or Mg can be of concern due to the well-known adverse effects of phytate in reducing the bioavailability of Zn or Mg in diets [32]. Other correlations in the study at hand were also found in other studies, such as those between Mn and Zn, Cu or P; Cu and Zn o Mn; Ca and Na or Mg [33].

However, no correlations associated with Fe were found. Gomez-Becerra et al. [32], Jiang et al. [33], and Vrček et al. [23] reported positive correlations associated with Fe with Zn or Mn. Mineral content, as well as their correlations, can be affected by environment and genotype. Minerals, in addition to being a source of nutrients for humans, have critical functions in the growth and development of plants [33].

### 3.2. Bread Results

In addition to the parameters analyzed in the flours, the different starch fractions (RS, RDS, and SDS) were also analyzed. We have no record that the nutritional composition of the flours and the resulting products were compared in other studies or when comparing different cultivation methods.

**Figure 4:**
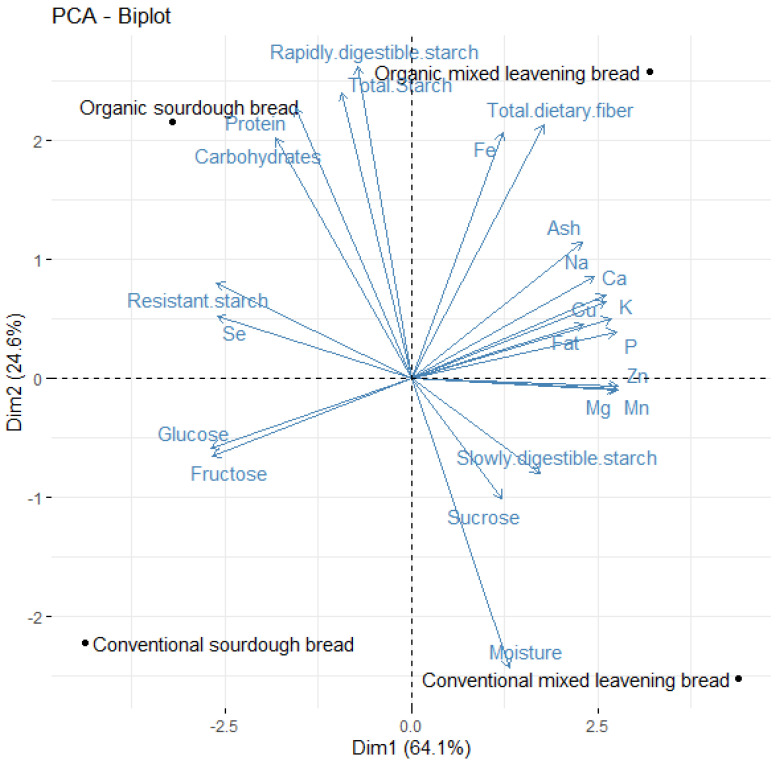
Principal component analysis (PC1 vs. PC2) describing the nutritional behavior among the breads.

In order to obtain an initial overview, a principal components analysis was carried out (Figure 4). While in the first dimension, a differentiated behavior between the mixing method and sourdough was observed (64.1% of the total variance explained), in the second one, a differentiated position was observed between the bread made with flour from organic and conventional cultivation (24.6% of the total variance explained). 

Thus, according to PCA analysis (PC1), bread made with the mixed method showed high values for elements (except Se) fiber, sucrose, and SDS. Sourdough bread had more TS, RS, RDS, protein, carbohydrates, glucose, and fructose. On the other hand, in PC2, organic bread showed a higher amount of protein, carbohydrates, fructose, TS, RS, RDS, total fiber, ash, Na, P, Fe, Zn, and Cu, and less moisture than conventional breads.

**Figure 5:**
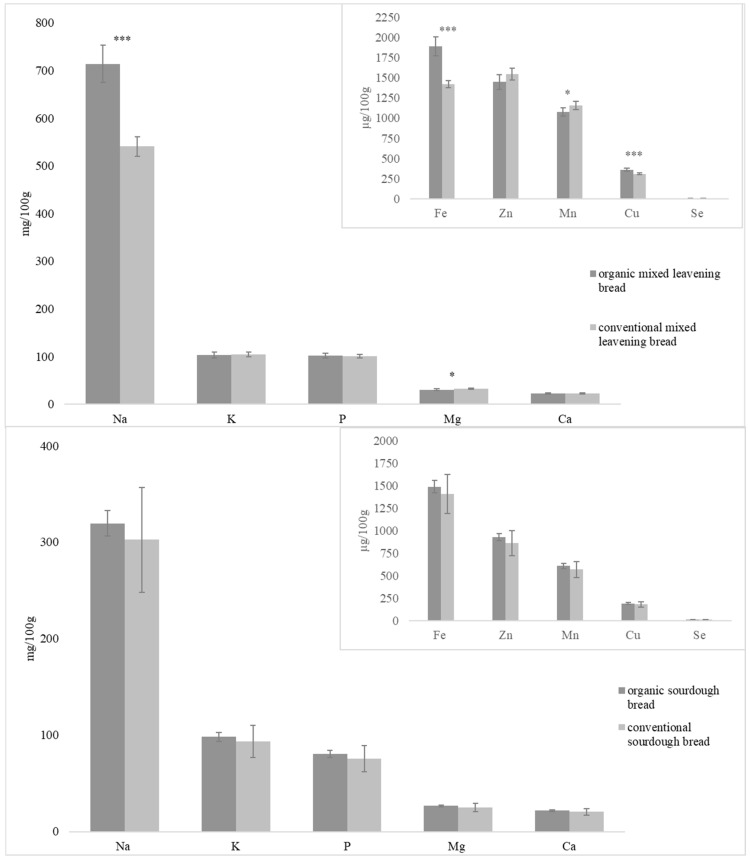
Mineral content of mixed leavening bread and sourdough bread made with flours from organically and conventionally grown wheat (* *p*-value < 0.05, ** *p*-value < 0.01 and *** *p*-value < 0.001).

For a more detailed analysis, a comparative test between the bread made with organic and conventional flour was carried out for each ferment (mixed method and sourdough). First of all, the values of the analyzed parameters decreased with respect to those presented by the starting flours, except for water, ash, and Na (Table 2 and Figure 5) because the elaboration process added salt and water to the flour. It was expected that adding water to the flour in the breadmaking process would reduce the content of protein, fat, carbohydrates, sugars, fiber, total starch, and minerals. Our results agreed with those reported by Abdel-Aal [34], where the protein content decreased in the wheat and spelt products studied when compared to the flours made with them.

The parameters analyzed in which significant differences in both types of bread were observed depending on the crop system were moisture, protein, carbohydrates, fructose, TS, RS, RDS, ash, Na, Mg, Fe, Mn, and Cu (Table 2 and Figure 5).

Organic bread presented less moisture (35.43 ± 0.03 and 37.18 ± 0.20 g/100 g for bread made with sourdough and bread made with the mixed method, respectively) than conventional bread (38.78 ± 0.02 and 40.74 ± 0.08 g/100 g for bread made with sourdough and bread made with the mixed method, respectively).

Organic breads contain more protein than conventional breads (Table 2). These data do not agree with the study by Dall’Asta et al. [35], in which they reviewed commercial products in Italy; for the ‘Pasta, rice and other cereals’ category, they found no differences as per the farming method. In the same way, for the ‘bread and substitutes’ category (analyzing wraps, crackers, breadsticks, bread, and rusks), they found no significant differences. In contrast, although they did not take into account such important factors as the variety of wheat or the growing conditions, Dello Russo et al. [36] compared the labeling of pasta sold in Italy and concluded that organic pasta had a lower protein concentration.

As observed in the protein content, the organic version of the bread also showed more carbohydrates than the conventional one. These results do not agree with other authors who studied the carbohydrate content in different products derived from cereals [35,36] since they did not find differences depending on the cultivation system (although, as previously mentioned, other factors were not taken into account). Carbohydrate-rich foods, such as wheat or bread, may contain the same amount of starch but have different rates and degrees of starch digestion and, consequently, different glycemic and insulinemic responses [10]. So, the analysis of starch digestibility in bread is of great interest from a nutritional point of view.

With both ferments, the organic bread presented higher amounts of TS, RS, and RDS. In the case of the SDS content, there is no clear influence of the culture system.

Nutritionally, high values of SDS and RS and lower values of RDS are required [8]. Conventional sourdough bread had a lower SDS content than organic sourdough bread, so this bread could have higher glycemic indexes than its organic counterpart. The conventional bread made by mixed leavening presented higher values of SDS than the organic bread and lower values of RDS; therefore, this bread presented lower glycemic indexes. Diets with lower glycemic indexes have been shown to decrease the long-term risk of type 2 diabetes and have been associated with good general health, especially during the aging process [37]. The values found in the present study were similar to those reported by Štěrbová et al. [9] and Simsek et al. [8], who studied wheat bread made from traditional and modern varieties. 

Conventional breads presented lower RS values than organic breads for both fermentation methods. Higher values were associated with a greater reduction in blood lipid levels and glycemic responses [10,11]. The RS content of the bread in this study presented values lower than 1%, which is an insignificant proportion, as explained by Goñi et al. [38].

Therefore, taking into account the other fractions, conventional bread made with mixed leavening could have a more beneficial impact on metabolism because it presented a lower RDS and a higher SDS value. SDS can be considered a slow-release carbohydrate, like the one that improves the control of blood sugar levels and whose benefits are related to diabetes control and satiety/food intake [37]. In the case of bread made with sourdough, better metabolic benefits were not clear because conventional bread presented lower concentrations of RDS than organic bread; at the same time, organic bread showed higher SDS concentrations than conventional bread.

The concentration of sugars (sucrose, glucose, and fructose) did not follow the same pattern in flour and in bread. In bread, there was more fructose than glucose and sucrose. In the ‘Caaveiro’ flours, 0.66 ± 0.02 g/100 g and 0.53 ± 0.01 g/100 g of sucrose were detected in organic and conventional flours, respectively, which, after its hydrolysis during bread fermentation, was hydrolyzed into glucose and fructose. For this reason, the fructose content was increased, and sucrose in the bread was reduced compared to flour (Table 1 and Table 2). In addition to presenting a different behavior from the flour, the bread presented a different behavior depending on the type of fermentation. The glucose was significantly higher in the conventional bread made with sourdough compared to the organic one, while the opposite behavior was observed in fructose, and no significant differences were found in the sucrose content. The bread made by the mixed method did not show significant differences in terms of glucose and fructose content, but the sucrose content was higher in conventional bread. The total sugar content was higher in the case of bread made with sourdough, maybe because the yeast added to the mixed leavening consumed more sugars present in the flour.

Following the same behavior as in flour, in organic bread, the fiber content was higher than in its conventional counterparts, although without significant differences (Table 1 and Table 2). In the study presented by Dello-Russo et al. [36], organic pasta sold on the Italian market had a higher fiber content than conventional pasta.

The bread followed the same trend as the flour in terms of the mineral concentration (K > P > Mg > Ca > Fe > Zn > Mn > Cu > Se), with the exception of Na, because, during bread making, NaCl was added, so it was the mineral that appeared at the highest concentration (Figure 5). In the case of sourdough bread, no differences were found between both farming systems; however, there were significant differences in Na, Mg, Fe, Mn, and Cu in mixed-leavening bread. The organic mixed leavening bread contains a higher concentration of Na, Fe, and Cu but a lower content of Mn and Mg. No significant differences were found for the rest of the minerals (K, Ca, P, Zn, and Se).

When regarding the amount of minerals in bread, the literature varies greatly. This is due to various factors such as the degree of refinement of the flour, the fortification of certain minerals such as Ca or Fe according to legal requirements, the composition of the soil, the amount of fertilizers applied, or the geographical location of the cultivars [39]. 

Most of the results in the present study agree with the results reported by Torrinha et al. [40] for wheat bread, except for the Mn and Cu contents that are higher in the present investigation. 

As indicated by different authors, minerals are necessary for human beings for the maintenance of health and the proper functioning of the human body. Some of the mineral elements are required in larger amounts (e.g., Ca, Mg, and K), while others, such as Fe, Zn, Cu, I, and Se, are required in smaller amounts [23]. 

Based on this, these breads could be a good source of P, K, Mg, Fe, Zn, and Cu, as suggested by Ciudad-Mulero et al. [41].

## 4. Conclusions

In the flour analysis, significant differences were found depending on the cultivation system. The organic flour presented higher moisture, fat, and sucrose content, while the content of total starch was higher in conventional flour. In addition, organic flour showed higher amounts of P, Na, and Cu and a lower content of Zn.

The global vision of the results obtained for bread for the two fermentation methods has shown that organic bread has a significantly higher content of protein, carbohydrates, total starch, resistant starch, rapidly digestible starch, ash, Na, P, Fe, and Cu content and less moisture than conventional bread. 

The bread made using flour from local ’Caaveiro’ wheat obtained by both cultivation systems allowed the production of quality Galician bread.

Despite the differences in the nutritional profile of flours and breads between the organic and conventional systems, ecological wheat production can achieve a quality product of the same level as the conventional one, independently of the fermentation process used, with lower incomes and a positive effect on the environment, which is increasingly appreciated by consumers.

In any case, it is necessary to delve deeper into the effect of the type of ferment and fermentation times on the nutritional profile of bread made with local wheat varieties.

## Figures and Tables

**Table 1:** Descriptive statistics of nutritional characteristics of flours from organically and conventionally grown wheat (mean ± standard deviation).

Parameter (g/100 g)	Organic	Conventional	Critical Significance Level
Moisture	13.26 ± 0.24	12.70 ± 0.14	***
Ash	0.62 ± 0.02	0.64 ± 0.01	ns
Protein	12.35 ± 0.27	12.22 ± 0.30	ns
Fat	1.65 ± 0.25	1.31 ± 0.23	**
Carbohydrates	71.99 ± 0.30	73.15 ± 0.34	ns
Sucrose	0.66 ± 0.02	0.53 ± 0.01	*
Glucose	0.13 ± 0.02	0.23 ± 0.00	ns
Fructose	0.03 ± 0.00	0.04 ± 0.00	ns
Total starch	66.50 ± 0.16	67.40 ± 0.20	*
Total dietary fiber	5.36 ± 0.16	5.12 ± 0.21	ns

ns, not significant, * *p*-value < 0.05, ** *p*-value < 0.01 and *** *p*-value < 0.001.

**Table 2:** Nutritional characteristics of bread made with organically and conventionally grown wheat.

Parameter (g/100 g)	Organic Sourdough	Conventional Sourdough	Significance Level	Parameter (g/100 g)	Organic Mixed Leavening	Conventional Mixed Leavening	Significance Level
Moisture	35.43 ± 0.03	38.78 ± 0.02	***	Moisture	37.18 ± 0.20	40.74 ± 0.08	***
Protein	9.18 ± 0.03	8.72 ± 0.18	*	Protein	8.87 ± 0.17	8.37 ± 0.09	*
Fat	0.18 ± 0.04	0.15 ± 0.04	ns	Fat	0.19 ± 0.02	0.20 ± 0.02	ns
Carbohydrates	54.05 ± 0.07	51.27 ± 0.22	***	Carbohydrates	51.53 ± 0.07	49.07 ± 0.11	***
Sucrose	0.07 ± 0.02	0.00 ± 0.00	ns	Sucrose	0.00 ± 0.00	0.14 ± 0.01	*
Glucose	0.21 ± 0.01	0.29 ± 0.01	*	Glucose	0.09 ± 0.0	0.08 ± 0.01	ns
Fructose	0.43 ± 0.03	0.41 ± 0.03	*	Fructose	0.20 ± 0.00	0.19 ± 0.00	ns
Total starch	49.57 ± 0.16	46.52 ± 0.16	***	Total starch	47.90 ± 0.30	46.14 ± 0.25	**
Resistant starch	0.94 ± 0.02	0.87 ± 0.02	**	Resistant starch	0.72 ± 0.02	0.65 ± 0.01	**
Rapidly digestible starch	47.83 ± 0.26	42.97 ± 0.41	***	Rapidly digestible starch	46.14 ± 0.29	42.06 ± 0.1	***
Slowly digested starch	1.14 ± 0.04	0.20 ± 0.04	***	Slowly digested starch	0.61 ± 0.04	2.26 ± 0.1	***
Total dietary fiber	4.16 ± 0.16	3.76 ± 0.16	ns	Total dietary fiber	4.41 ± 0.19	4.09 ± 0.05	ns
Ashes	1.15 ± 0.02	1.07 ± 0.01	**	Ashes	2.23 ± 0.09	1.61 ± 0.04	***

ns, not significant, * *p*-value < 0.05, ** *p*-value < 0.01 and *** *p*-value < 0.001.

## Data Availability

The original contributions presented in the study are included in the article, further inquiries can be directed to the corresponding author.
